# Risk factors for receiving both scleral buckle and glaucoma drainage device in children

**DOI:** 10.1038/s41598-024-76352-7

**Published:** 2024-10-25

**Authors:** Mariana Abi Karam, Arjun Sharma, Ashley Lopez-Canizares, Piero Carletti, Elizabeth A. Vanner, Audina M. Berrocal, Ta Chen Chang

**Affiliations:** Bascom Palmer Eye Institute, University of Miami Miller School of Medicine, 900 NW 17th Street .450N, Miami, FL 33136 USA

**Keywords:** Pediatric eyes, Scleral buckle, Glaucoma drainage device, Ocular trauma, Outcomes research, Paediatric research

## Abstract

We conducted a retrospective review of patients (< 18 years) between 2013 and 2021 to determine risk factors of pediatric patients receiving a glaucoma drainage device (GDD) needing a scleral buckle (SB) and vice versa. The first population underwent an SB placement with no prior GDD. The second underwent a GDD implantation with no prior SB. We analyzed 133 eyes in the SB-first group. The common indications were retinal detachment associated with a syndrome/disease (41.2%), blunt trauma (21%), and non-syndromic pathologic myopia (11%). Twelve eyes (9%) required subsequent GDD implantation with a mean time to reoperation of 19.73 months (median 2.3 months). Eyes with trauma had a 5-fold risk (hazard ratio [HR] 4.81, *p* = 0.0069) of requiring a subsequent GDD. Each additional glaucoma medication before initial SB surgery increased the risk by 1.81 (*p* = 0.0096). 135 eyes were in the GDD-first group. Four eyes (3%) required a subsequent SB. The mean time to reoperation was 17 months (median 9.2 months). There was a statistically significant association with trauma (HR 12.3, *p* = 0.0341) only. Pediatric eyes with a history of trauma and/or multiple glaucoma medications before intervention have a high risk of requiring both an SB and GDD.

**Supplementary Information:**

The online version contains supplementary material available at 10.1038/s41598-024-76352-7.

## Introduction

A scleral buckle (SB) is an encircling band surgically placed around the eye in the sub-Tenon space, either alone or in combination with vitrectomy, as prophylaxis against or as a means to treat retinal detachment (RD)^1^. A glaucoma drainage device (GDD), which is also placed in the sub-Tenon space, is often required in cases of refractory glaucoma. Pediatric eyes with SB may develop elevated intraocular pressure (IOP) and have an increased risk of requiring a subsequent GDD placement^2,3^. Meanwhile, glaucomatous pediatric eyes share many features with eyes at risk of developing an RD, such as axial myopia, trauma history, aphakia, and diabetes among others^2,3^. Furthermore, GDD procedures may have vision-threatening vitreoretinal complications, such as serous choroidal effusion, suprachoroidal hemorrhage, and RD^4^. Since both SB and GDD occupy the same anatomic (sub-Tenon) space, sequential placement of these implants can pose significant challenges. In eyes with previously placed SB, conjunctival scarring requires extensive dissection and decreases the size of the potential space in which GDD is placed, while the scarred conjunctiva increases the risk of post-operative hardware exposure^5^. In eyes with previously placed GDD that requires SB, to allow proper scleral indentation, the GDD often needs to be explanted and re-implanted. To optimize care for these challenging patients, identifying the risk factors for needing a subsequent SB when a GDD is being implanted (and vice versa) would be helpful in planning concurrent surgeries in the appropriate patients. However, the literature discussing the simultaneous management of RD and glaucoma is rare overall, and is even more scarce for the pediatric population^2^, focused mostly on the management of acute ocular hypertension or glaucoma after vitreoretinal surgeries^2^.

Our study aims to determine which pediatric eyes that undergo one type of implant (SB or GDD) is at risk of needing the other implant, to identify high-risk patients who require multiple sub-Tenon implants.

## Methods

University of Miami Institutional Review Board/Ethics Committee approval was obtained for this study and the need for informed consent was waived. In addition, this study protocol adhered to the tenets of the Declaration of Helsinki and complied with the Health Information Portability and Accountability Act. We identified all patients who had undergone either SB or GDD implantations between 2013 and 2021 using a combination of International Classification of Diseases and Current Procedural Terminology codes. All available medical records were reviewed. Entries were included for analysis if the age at the time of the procedure was < 18 years and were excluded if the eye had a pre-existing sub-Tenon or subconjunctival implant of any type, or if the SB and GDD were implanted on the same date. Preoperative data collected included patient demographics, systemic and ocular comorbidities/syndromes, history of ocular trauma, glaucoma diagnosis (if any), type of retinal detachment (if any), best corrected visual acuity (BCVA), IOP, and number of glaucoma medications. Postoperative data collected included the timing of the GDD or SB implantation (if any). At each postoperative visit, BCVA, IOP, number of glaucoma medications, retina status, complications, and subsequent procedures were assessed. The eyes that underwent a SB intervention with no prior GDD were categorized as the “SB-first group,” while eyes that underwent a GDD implantation with no prior SB were categorized as the “GDD-first group.” Survival analysis was performed with subsequent GDD (for SB-first group) or SB (for GDD-first group) implantation as the failure event. Snellen BCVA was transformed to logMAR to perform statistical analyses.

Descriptive statistics for categorical variables include counts and percentages and for continuous variables include means and standard deviations. Time to reoperation was assessed using Kaplan-Meier survival analysis to produce survival curves and Cox proportional hazard regression paired frailty model to account for some subjects having both eyes in the sample. All analyses were done using SAS version 9.4 software (SAS Institute, Cary, NC, USA). A p-value *≤* 0.05 was considered statistically significant.

## Results

The baseline characteristics of each study population are presented in Table 1. The SB-first group included 133 eyes and the GDD-first group included 135 eyes. The average age of patients in the SB-first group and GDD-first group was 11.2 ± 4.5 and 7.49 ± 5.46 years, respectively. GDDs consisted of Baervedlt Glaucoma Implants (both models 250 and 350, Johnson & Johnson Vision, Jacksonville, FL, United States) and Ahmed Glaucoma Valves (model FP7, New World Medical, Inc., Rancho Cucamonga, CA, United States). GDDs were placed without the use of antimetabolites in either the superotemporal or the inferonasal quadrants, the decision guided by the relative density of conjunctival scarring (if any) in each quadrant and the presence of silicone oil in the intended tube tip location. All scleral buckles were encircling. In the SB-first group, 35% of patients had an associated syndrome compared with 15% of patients in the GDD-first group, while the GDD-first group had longer follow-up (40.9 +/− 28.5 months vs. 31.6 +/− 29.4 months, *p* = 0.0091). Associated syndromes/diseases in the SB-first group included familial exudative vitreoretinopathy, Stickler syndrome, Marfan syndrome, Sturge-Weber syndrome, Knobloch syndrome, X-linked retinoschisis, and Coats disease. Associated syndromes/diseases in the GDD-first group included aphakia/pseudophakia, Sturge-Weber syndrome, and Axenfeld-Rieger syndrome. In the SB-first group, 22% of eyes had a history of ocular trauma compared with 3% of eyes in the GDD-first group. The mean IOP in the SB-first group before the initial surgery was 15.1 ± 4.67 mmHg with patients using an average of 0.17 ± 0.65 pressure-lowering medications. In the GDD-first group, the mean IOP before surgery was 29.9 ± 10.7 mmHg with patients using an average of 3.12 ± 1.23 pressure-lowering medications.

**Table 1:** Baseline characteristics of scleral buckle and Glaucoma drainage device groups.

	SB Group (*n* = 133 eyes of 121 patients)	GDD Group (*n* = 135 eyes of 115 patients)
Reoperation, n (%)		
No	121 (91)	131 (97)
Yes	12 (9)	4 (3)
Age (years)		
Mean ± SD	11.2 ± 4.5	7.49 ± 5.46
Sex, n (%)		
Female	46 (35)	56 (41)
Male	87 (65)	79 (59)
Laterality, n (%)		
Right	63 (47)	69 (51)
Left	70 (53)	66 (49)
Associated syndrome/disease, n (%)		
No	86 (65)	115 (85)
Yes	47 (35)	20 (15)
Associated trauma, n (%)		
No	104 (78)	131 (97)
Yes	29 (22)	4 (3)
Glaucoma medications before first procedure		
Mean ± SD	0.17 ± 0.65	3.12 ± 1.23
LogMAR VA before first procedure		
Mean ± SD	1.23 ± 0.88	1.50 ± 0.93
IOP (mmHg) before first procedure		
Mean ± SD	15.1 ± 4.67	29.9 ± 10.7

SB, scleral buckle; GDD, glaucoma drainage device; IOP, intraocular pressure; LogMAR, logarithm of the minimal angle or resolution; VA, visual acuity; SD, standard deviation.

**Figure 1:**
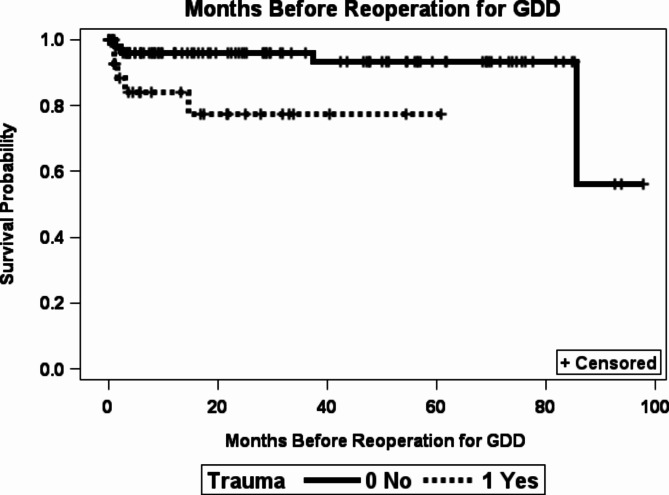
Scleral bucklefirst eyes survival curve with subsequent glaucoma drainage device (GDD) implantation defined as the failure event.

A total of 133 eyes (46 [34.6%] female) were in the SB-first group. The most common indications for initial scleral buckle surgery were RD associated with a syndrome or disease (41%), blunt trauma (21%), and non-syndromic pathologic myopia (11%). Over the study period with a mean follow-up time of 31.6 ± 29.4 months and median of 22.5 months (Interquartile range (IQR) = 5.97, 54.3 months), 12 eyes (9%) had required GDD implantation; 121 eyes (91%) did not require a GDD implantation. The mean time to GDD implantation was 19.74 months with a median of 2.3 months. Blunt trauma increased the risk of needing future reoperation by almost 5-fold (hazard ratio [HR] 4.81, *p* = 0.0069) (Fig. 1). Each additional pressure-lowering medication prior to the initial SB surgery increased the risk of requiring GDD by almost 2-fold (HR 1.81, *p* = 0.0096). There was a marginally significant association between each 1.00 logMAR unit decrease of pre-operative VA and the need for GDD implantation (HR 1.82, *p* = 0.067). Neither gender nor having a syndrome or disease demonstrated a statistically significant association with the need for future GDD implantation.

A total of 135 eyes (56 [41%] female) were in the GDD-first group. The most common indications for the initial GDD surgery were primary congenital or juvenile open-angle glaucoma (37%), glaucoma associated with acquired conditions or following cataract surgery (33%), and glaucoma associated with non-acquired ocular anomalies, systemic diseases, or syndrome (18%). Over the study period with a mean follow-up time of 40.9 ± 28.5 months and median of 43.4 months (IQR = 15.6, 64.8 months), 4 eyes (3%) had undergone a SB procedure; 131 eyes (97%) did not require a SB procedure. The mean time to reoperation was 17 months with a median of 9.2 months. The only statistically significant risk factor for requiring a SB following initial GDD was with trauma, resulting in a 12-fold increase in the need for a SB (HR 12.3, *p* = 0.0341) (Supplemental Fig. 1). In addition, better visual acuity prior to the initial surgery GDD placement was associated with a decreased likelihood of needing a SB (HR 0.36, *p* = 0.0278). No patients with a syndrome/disease required SB after GDD implantation, therefore there was no association between having a syndrome and requiring reoperation (*p* = 0.407).

## Discussion

Many pediatric eyes with retinal detachment may develop glaucoma, while many pediatric eyes with glaucoma are at high risk for retinal detachment. The surgical management of concurrent pediatric retinal diseases and glaucoma is complex and often conflicting, with the surgical treatment of one sometimes limiting future treatment options for the other. SB and GDD are among the most important procedures in the management of pediatric retinal detachment and pediatric glaucoma, respectively. To our knowledge, this is the first study to examine the risk factor of pediatric eyes requiring both implants. Our results suggest that pediatric eyes with a history of blunt trauma (pending either GDD or SB) have a high risk of needing the other implant, while eyes requiring pressure-lowering medications before scleral buckling have a high risk of requiring a future GDD.

In adults, it is well known that eyes with retinal detachment have a higher incidence of glaucoma^6^. When a RD is managed with a SB, sequential insertion of a GDD presents technical challenges to the surgeon due to conjunctival scarring and possible epithelial ingrowth into the fibrous capsule increasing the risk of a wound leak^7,8^. Alternatively, some have proposed the modified Schocket procedure as a means of lowering IOP, although the outcome compared to a GDD remains uncertain^9–11^. Despite these challenges, Scott et al. described a 16-patient series of Baerveldt glaucoma implant placement in adult eyes with preexisting SBs leading to effective IOP control and minimal postoperative complications^12^. Zhang et al. also examined the post-operative course of two-step surgery in 17 eyes with preexisting SBs who underwent 250 mm^2^ Baerveldt implants. Nearly 25% of eyes in their study experienced conjunctival dehiscence, which required surgical revision within one month, while 2 eyes (12%) developed persistent exposure resulting in GDD explantation^13^. Lima and colleagues reported a series of 30 patients that underwent simultaneous Baerveldt glaucoma implantation and SB placement for uncontrolled IOP and retinal detachment. This group found successful IOP control and reduction in the number of glaucoma medications in eyes that required a second-stage tube without tube migration, fibrous tissue obstruction, or any complication related to combined surgery, which supports the notion of simultaneous SB/GDD implantation in the appropriate eyes^2^.

The strongest predictor of SB-first eyes needing a GDD in our cohort was ocular trauma, which increased the likelihood by nearly 5-fold. Prior literature reported a 6-month incidence of posttraumatic glaucoma of 3.4% following blunt ocular injury^14^. Another large study found the presence of hyphema, corneal injury, and visual acuity < 20/200 as independent risk factors for surgical intervention in secondary glaucoma from trauma^15^. In pediatric eye trauma, predictors of uncontrolled glaucoma needing surgical intervention include severe anterior chamber reaction and corneal edema^16^. Glaucoma develops after trauma by several mechanisms, including direct damage to the trabecular meshwork, bleeding, inflammatory scarring, and lens particles obstructing outflow^17,18^. In the appropriate patient who is undergoing scleral buckling for retinal detachment after blunt trauma, given the high risk of secondary glaucoma and technical difficulties with implanting a GDD following an SB, a simultaneous SB and stage 1 GDD may be considered.

Each additional pressure-lowering medication before SB surgery increased the likelihood of needing subsequent GDD by nearly 2-fold in our study population. Elevated IOP has been described as a complication following SB, especially in patients < 40 years old^3^. We also found a marginally significant association between having worse visual acuity preoperatively and increased odds of needing a GDD.

Placement of an SB in an eye with a pre-existing GDD necessitates the removal of the GDD to achieve proper scleral indentation and support of the vitreous base. Surgical techniques to circumvent this problem have been reported in adults. For example, Kim and Smiddy described a technique to incorporate a preexisting Baerveldt glaucoma implant into an SB using a 240 silicone band, although it is unclear how the scleral indentation is achieved over the GDD plate^19^. In our cohort of 135 GDD-first eyes, only 4 eyes (3%) required an SB, with trauma being the greater risk factor of an eye requiring subsequent SB. The low incidence of GDD-first eyes needing subsequent SB may be due to the cohort’s younger age, such that the formed vitreous may tamponade rather than dissect into retinal holes or tears.

Our study has several limitations. The retrospective nature and lack of randomization in the study design may introduce biases. Specifically, in our referral center cohort, many eyes in the SB-first group lacked detailed information regarding prior surgeries, hence we cannot directly investigate whether a history of pre-SB, conjunctival-involving procedures is a risk factor for requiring a subsequent GDD. However, we have investigated the variables of “associated diseases/syndromes” and “associated trauma” (Table 1) being risk factors for the SB-group eyes receiving a subsequent GDD, which would adequately capture any pre-SB procedures. The relatively small number of patients who required subsequent implants in both cohorts, particularly the GDD-first group, may limit the power of our results. In addition, some patients have both eyes in the study, and this was not accounted for in the Kaplan-Meier survival analysis, despite being adjusted in the Cox proportional hazard regressions. Despite these limitations, our study is the largest series of its kind in a subspecialty, and the findings may guide clinicians to optimize their decision-making when managing children who require SB and/or GDD. In summary, in our large series, eyes undergoing either GDD or SB with a history of blunt trauma may benefit from the concurrent placement of both implants, while eyes pending SB with a history of anti-glaucoma medication use may benefit from the concurrent implant of a GDD.

## Electronic supplementary material

Below is the link to the electronic supplementary material.

Supplementary Material 1

## Data Availability

The datasets generated during and/or analyzed during the current study are available from the corresponding author on reasonable request.
