# Supplementary material for: Risk factors for receiving both scleral buckle and glaucoma drainage device in children
Source: Sci Rep. 2024 Oct 25;14:25300. doi: 10.1038/s41598-024-76352-7 (PMC11511900; doi:10.1038/s41598-024-76352-7)
Supplement: Supplementary file 1 — Supplementary Material 1 [file 41598_2024_76352_MOESM1_ESM.pdf]

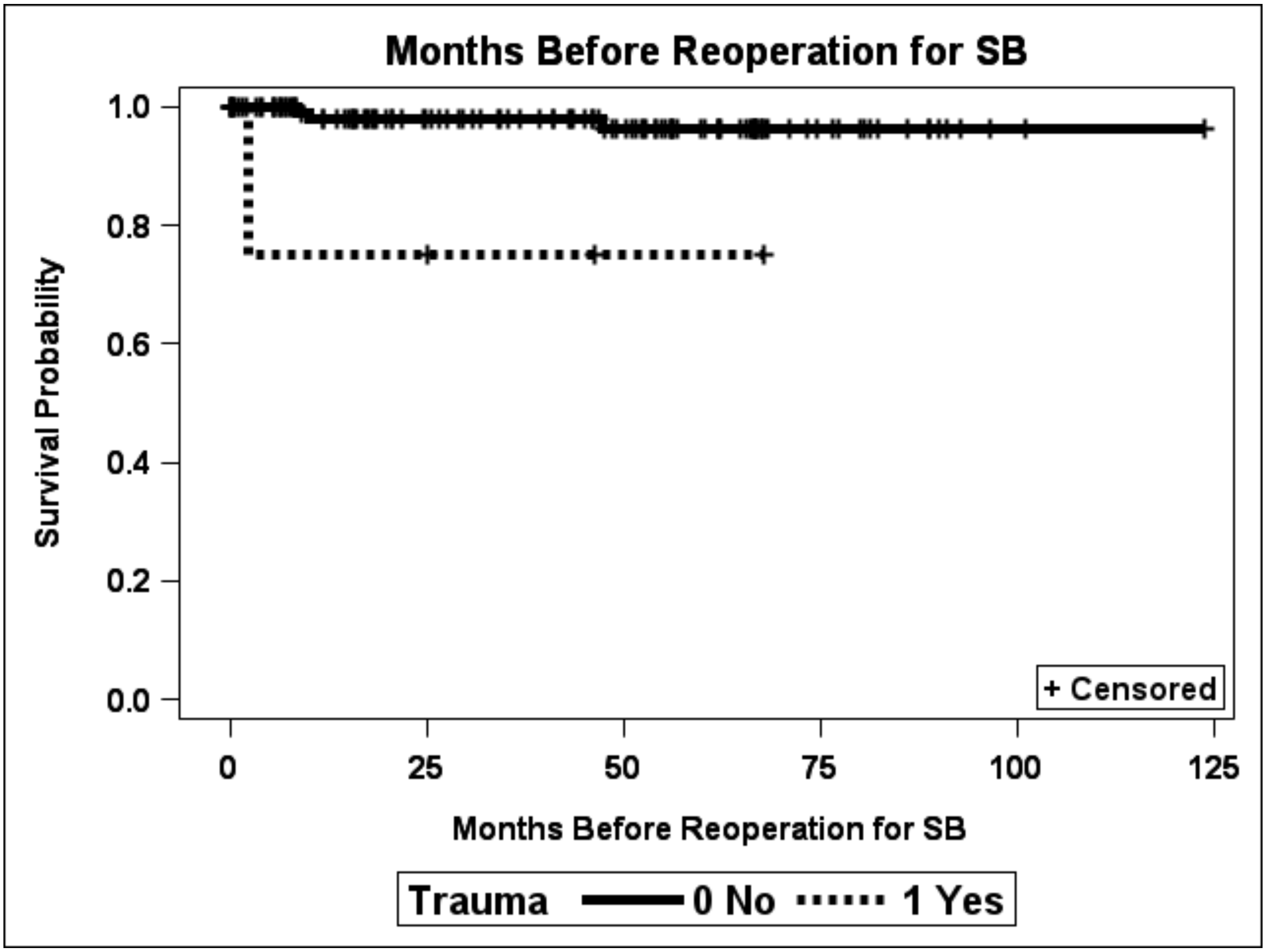

Supplemental Figure 1. Glaucoma drainage device first survival curve with subsequent scleral buckle (SB) defined as the failure event.
